# Cloning, expression, and characterization of a *Coxiella burnetii* Cu/Zn Superoxide dismutase

**DOI:** 10.1186/s12866-015-0430-8

**Published:** 2015-05-12

**Authors:** Robert E Brennan, Katalin Kiss, Rachael Baalman, James E Samuel

**Affiliations:** Department of Biology, University of Central Oklahoma, 100 North University Drive, Edmond, OK USA; American Type Culture Collection, Manassas, VA USA; Department of Medical Microbiology and Immunology, Texas A & M University System Health Science Center, College Station, TX USA

**Keywords:** *Coxiella*, Copper-zinc superoxide dismutase, Q fever

## Abstract

**Background:**

Periplasmically localized copper-zinc co-factored superoxide dismutase (SodC) enzymes have been identified in a wide range of Gram-negative bacteria and are proposed to protect bacteria from exogenously produced toxic oxygen radicals, which indicates the potential significance of a *Coxiella burnetii* SodC.

**Results:**

Assays for SOD activity demonstrated that the cloned *C. burnetii* insert codes for a SOD that was active over a wide range of pH and inhibitable with 5 mM H_2_O_2_ and 1 mM sodium diethyldithiocarbamate, a characteristic of Cu/ZnSODs that distinguishes them from Fe or Mn SODs. The *sod*C was expressed by *C. burnetii*, has a molecular weight of approximately 18 kDa, which is consistent with the predicted molecular weight, and localized towards the periphery of *C. burnetii*. Over expression of the *C. burnetii sod*C in an *E. coli sodC* mutant restored resistance to H_2_O_2_ killing to wild type levels.

**Conclusions:**

We have demonstrated that *C. burnetii* does express a Cu/ZnSOD that is functional at low pH, appears to be excreted, and was able to restore H_2_O_2_ resistance in an *E. coli sod*C mutant. Taken together, these results indicate that the *C. burnetii* Cu/ZnSOD is a potentially important virulence factor.

## Background

*Coxiella burnetii*, the etiologic agent of Q fever, is an obligate intracellular bacterium that replicates within the phagolysosome of monocytes/macrophages. The ability to survive in the harsh environment of the phagolysosome may require the subversion of macrophage microbicidal mechanisms. Several enzyme systems potentially required to survive in the phagolysosomal compartment, such as catalase, cytoplasmically localized superoxide dismutase (SOD), and acid phosphatase have been partially characterized [1,2]. Catalase and SOD activities were detected in *C. burnetii* whole cell lysates and were demonstrated to be maximally active at neutral pH suggesting that these enzymes were cytoplasmically localized and may be involved in detoxifying endogenously generated oxygen radicals [1]. Additionally, Heinzen *et al.* were able to clone a *C. burnetii* SOD and functionally complement an *E. coli sodA sodB* double mutant [2]. This *C. burnetii* SOD was demonstrated to be homologous to known iron-containing SODs. Baca *et al.* also demonstrated that supernatants from high-speed centrifugation of sonicated *C. burnetii* contained acid phosphatase activity that was optimally active at low pH, localized to the periplasmic space of *C. burnetii*, and was capable of inhibiting superoxide anion production by stimulated human neutrophils, suggesting that this enzyme may prevent killing of the bacteria during uptake by inhibiting the respiratory burst [3,4].

The *C. burnetii* genomic database from TIGR predicted a putative Cu/Zn SOD with a signal sequence (CBU 1822). Recently, Stead *et al.* reported the presence of a putative SodC protein in supernatants from *C. burnetii* cultured in acidified citrate cysteine media using a FLAG-tag assay, which indicates the secreted nature of the SOD [5]. Periplasmically localized Cu/Zn SOD enzymes have been identified in a wide range of Gram-negative bacteria and are proposed to protect bacteria from exogenously produced toxic oxygen radicals, which indicate the potential significance of a *C. burnetii* Cu/Zn SOD. For example, the survival of *Mycobacterium tuberculosis* and *Salmonella typhimurium sodC* mutants were reduced by 90% and 5 fold, respectively, compared to wild type when exposed to exogenous superoxide anion [6,7]. Additionally, Strohmeier Gort *et al.* reported that an *Escherichia coli sodC* mutant was more sensitive to hydrogen peroxide killing during stationary phase than wild type and were able to restore resistance to hydrogen peroxide killing through complementation [8]. Here we describe the cloning, expression, and characterization of a *C. burnetii* Cu/Zn SOD.

## Results

### Demonstration of CuZnSOD activity

**Figure 1:**
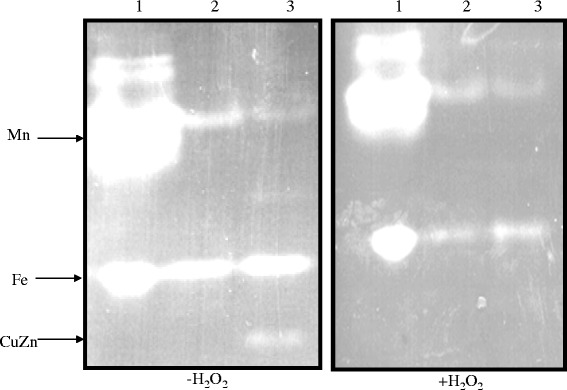
SOD activity gels. *C. burnetii* SodC activity was assayed in crude cell extracts of *E. coli* AS454 carrying *C. burnetii* sodC (pREB102) loaded on 12% native-PAGE gels and stained for SodC activity without and in presence of the Cu/ZnSOD inhibitor H_2_O_2_. Lanes: 1: *E. coli* MnSOD (9 μg) Sigma-Aldrich and FeSOD (2.5 μg) Sigma-Aldrich, 2: 30 μg of *E. coli* AS454/pREB102 lysate, 3: 30 μg *E. coli* AS454/pREB102 lysate after 4 h induction with 2% arabinose. - H_2_O_2_ and + H_2_O_2_ gels are identical; however, prior to being stained for SOD activity, the + H_2_O_2_ gel was soaked in 5 mM H_2_O_2_ for 1 hr.

**Figure 2:**
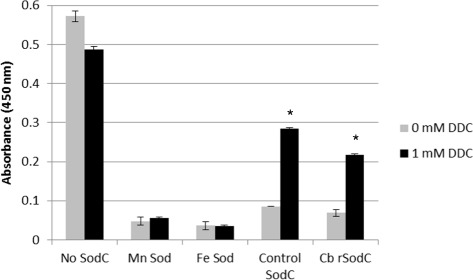
Sodium Diethyldithiocarbamate (DDC) inhibition of SOD activity. To further support the copper-zinc nature of the recombinant *C. burnetii* SOD, SOD activity assays were performed in the presence or absence of 1 mM DDC. Results represent the mean and standard deviation of three independent experiments. Approximately one unit of each SOD was used in the assay. (*) indicates a statistically significant difference between the 0 mM DDC and 1 mM DDC. Significant differences were determined using an unpaired two-tailed *t*-test.

**Figure 3:**
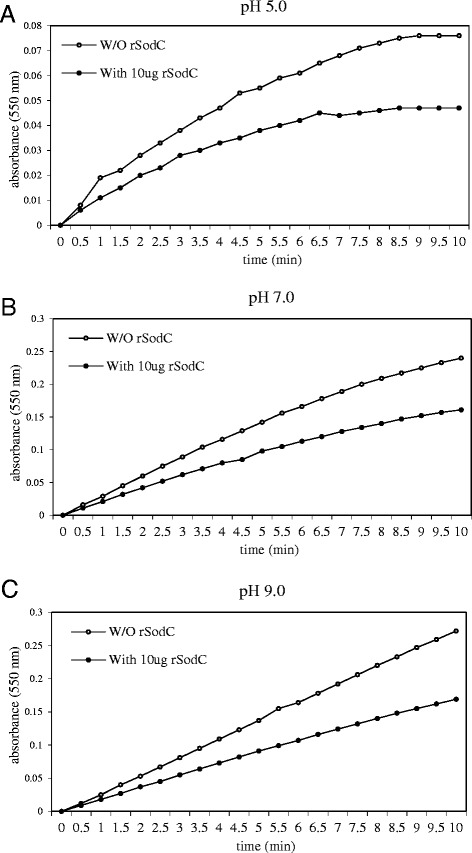
Effect of pH on CuZnSOD acitivity. Purified recombinant *C. burnetii* CuZnSOD was assayed for its ability to inhibit the reduction of cytochrome c at pH 5.0, **(A)**; pH 7.0, **(B)**; and pH 9.0, **(C)**. Open circles represent the reduction of cytochrome C in the absence of SOD. Closed circles represent the reduction of cytochrome C in the presence of SOD.

A *C. burnetii sodC* gene was cloned into pBAD, expressed in AS454 *E. coli* cells and assayed for enzymatic activity using SOD activity gels and a cytochrome C reduction assay. Cu/ZnSODs are inhibitable with millimolar concentrations of H_2_O_2_ and sodium diethyldithiocarbamate (DDC) [9,10]. This characteristic can be exploited in a polyacrylamide gel system to distinguish Cu/ZnSODs from Fe or Mn co-factored SODs. Over-expression of the cloned *C. burnetii sodC* gene by AS454(pREB102) revealed that Cu/ZnSOD activity was inhibited by 5 mM hydrogen peroxide (H_2_O_2_) but the Mn co-factored *E. coli* SOD activity was not. Slight inhibition of the Fe co-factored *E. coli* SOD also occurred, which is consistent with H_2_O_2_ exposure (Figure 1). DDC, which is known to be a specific inhibitor of Cu/Zn co-factored SODs, significantly (p < 0.05) inhibited the activities of both the recombinant *C. burnetii* SOD and the control bovine erythrocyte Cu/Zn co-factored SOD, but not the Mn or Fe co-factored SODs (Figure 2). Without inhibitor, the recombinant *C. burnetii* SOD demonstrated similar activity as the control SOD. In the absence of SOD, the DDC did lower the baseline absorbance values but did not impact the ability to determine the nature of the rCbSOD ion co-factor. These data confirm that CBU1822 indeed encodes a typical Cu/ZnSOD. The lack of detection of *E. coli* Cu/ZnSOD activity in the lysates from the non-induced AS454 (pREB102) is indicative of its relatively low concentration compared to that of Fe and Mn co-factored SODs [10]. Due to the acidic nature of the environment in which *C. burnetii* replicates, we predicted that the *C. burnetii* Cu/ZnSOD would be active at low pH. The activity of purified recombinant *C. burnetii* Cu/ZnSOD was assessed at three different pH values, 5.0, 7.0, and 9.0, using a xanthine/xanthine oxidase reduction of cytochrome C assay. Activities of superoxide dismutase enzymes in this assay are determined by their ability to inhibit the reduction of cytochrome C. SodC was active at all three pH values (Figures 3 A, B, and C). After 10 minutes there was approximately 45% inhibition of cytochrome C reduction by SodC at pH 9.0, 40% inhibition of cytochrome C reduction by SodC at pH 7.0 and 50% inhibition of cytochrome C reduction by SodC at pH 5.0. Taken together these data demonstrate that *C. burnetii* encodes for a copper zinc-dependent SodC, which is active at a low pH.

### Expression and subcellular localization of *C. burnetii* SodC

**Figure 4:**
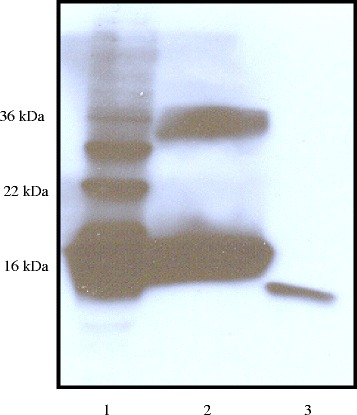
Western blot analysis of *C. burnetii* cell lysate. *C. burnetii* whole cell lysates were probed with polyclonal rabbit antiserum against rSodC. The polyclonal rabbit sera reacted with an approximately 18-kDa antigen in TOP10pREB102 induced with 2% arabinose (lane 1), purified rSodC (lane 2), and *C. burnetii* Nine Mile phase I cell lysates (lane 3).

**Figure 5:**
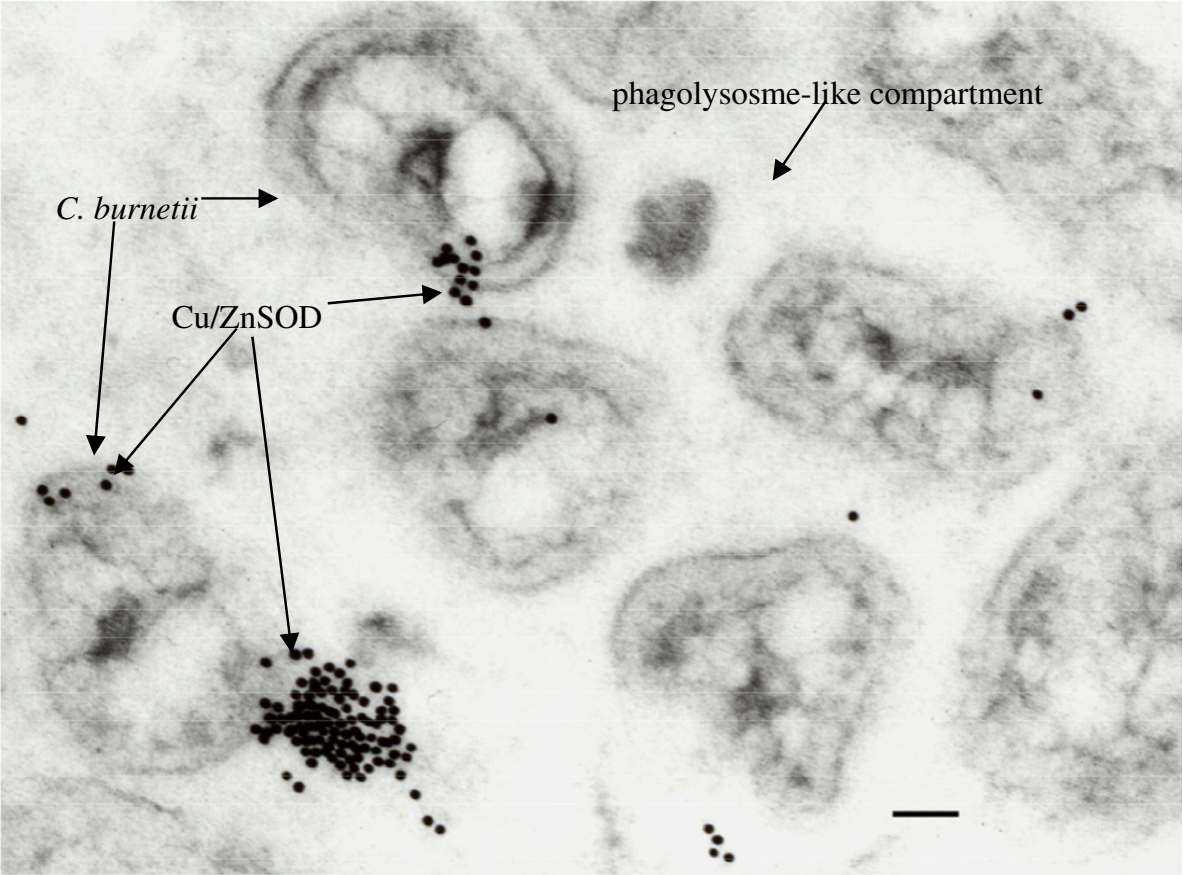
Immunogold electron microscopy of *C. burnetii*. Image shows localization of the *C. burnetii* Cu/ZnSOD. Several Cu/ZnSOD excreting *C. burnetii* are visible inside of a phagolysosome-like compartment. Bar=0.1 μm.

To evaluate the expression of *sod*C by *C. burnetii* monospecific polyclonal rabbit antiserum was generated against rSodC. Western blot analysis of *C. burnetii* whole cells lysates with this antiserum demonstrated cross reactivity with a single protein of approximately 15-kDa, which is in agreement with the predicted size of the *C. burnetii* SodC (Figure 4, lane 3). The slightly lower molecular weight of the *C. burnetii* SodC compared to the rSodC in lane two is likely due the removal of the approximately 2 kDa signal sequence during the secretion process and the lack of the approximately 1 kDa polyhistidine fusion tag present on the rSodC. The additional bands in lane one of Figure 3 are likely due to antibodies in the polyclonal rabbit antiserum raised against residual *E. coli* proteins still present in the purified rSodC preparation used to immunize the rabbit or due to previous environmental exposure of the rabbits to *E. coli*. In lane two of Figure 3, the purified rSodC appears to migrate as two bands, one band visible at approximately18 kDa which is in line with the predicted size, and a second band visible at approximately 36 kDa. The larger 36 kDa band is possibly the result of recombinant protein aggregates that have formed due to the presence of imidazole in the elution buffer [11]. It is also possible that the presence of the larger band is due to antibodies in the polyclonal rabbit antiserum raised against residual *E. coli* proteins still present in the purified rSodC preparation used to immunize the rabbit or due to previous environmental exposure of the rabbits to *E. coli* such as in lane one. Given that the antibody only reacts with a single band of the right size in the *C. burnetii* extract (Figure 4, lane 3), and the rSodC demonstrates Cu/Zn SOD activity (Figures 1 and 2), we are confident that the 18 kDa band in lane two of Figure 4 is rSodC. Localization of the *C. burnetii* SodC was achieved using immunoglold transmission electron microscopy on *C. burnetii*, Nine Mile, (RSA493) infected L929 murine fibroblast cells. *C. burnetii* SodC was shown to localize primarily towards the outer membrane and appears even to be excreted into the phagolysosome (Figure 5). Immunogold transmission electron microscopy was carried out on non-infected L929 murine fibroblasts as well and demonstrated no immunogold labelling with the rabbit anti-rSodC antibody (data not shown).

### Complementation of an *E. coli sodC* mutant with recombinant *C. burnetii* SodC

**Figure 6:**
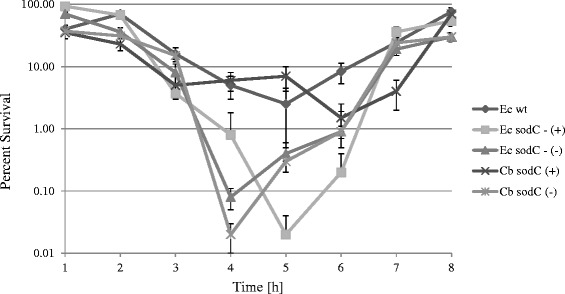
Complementation of *E. coli sod*C mutant. *E. coli* AS454 (Ec *sod*C-) with *C. burnetii sod*C (Cb *sod*C) under induced (+) and uninduced (-) conditions. Percent survival was determined after treatment with 2 mM H_2_O_2_ at 45 min intervals and compared to wild-type *E. coli* AN387 (Ec wt). The symbols and error bars represent the averages and standard deviations of three replicates.

The lack of a well-established genetics system for *C. burnetii* requires the use of heterologous cloning to assess *C. burnetii* gene function. pREB102 was transformed into an *E. coli sodC* mutant that was previously demonstrated to be highly susceptible to H_2_O_2_ killing in stationary phase [8]. The ability of the recombinant SodC to compliment an *E. coli sodC* mutant was assessed by comparing sensitivities to exogenously added H_2_O_2_. When exposed to 2 mM H_2_O_2_ the wild type (AN387) strain was sensitive and the *sodC* mutant (AS454) strain was highly sensitive to killing during the onset of stationary phase. Cells became resistant to H_2_O_2_ once again approximately 3 h later (Figure 6). This observation is in agreement with what Strohmeier-Gort *et al.* had previously reported for these strains [8]. Strain AS545 complemented with pREB102, when induced with 2% arabinose, demonstrated resistance to H_2_O_2_ killing similar to the wild type strain. Growth rates for all three strains were similar (data not shown).

## Discussion

There is mounting evidence that demonstrates the importance of Cu/ZnSOD enzymes in bacterial protection against oxidative killing. In fact, inactivation of the *sodC* gene has been found to cause attenuation of virulence in a wide variety of pathogenic bacteria [8,12-15]**.** The potential importance of a Cu/ZnSOD for the intracellular survival of *C. burnetii* is apparent by the obligate intracellular nature of this pathogen. In an effort to begin to characterize the potential role of this enzyme in *C. burnetii* intracellular survival a 570-bp region containing the signal sequence of the *C. burnetii sodC* gene was PCR amplified, cloned into pBAD-TOPO, and expressed as a fusion protein in TOP10 *E. coli* cells. The copper-zinc nature of this SOD was demonstrated by its inhibition by DDC and H_2_O_2_ using xanthine oxidase and native PAGE as demonstrated for other bacterial Cu/ZnSOD enzymes [10,16,17]. Several previously characterized Cu/ZnSOD enzymes contain signal sequences and were demonstrated to localize to the periplasmic space [10,14,15,18] leading to the hypothesis that these enzymes aid in the detoxification of superoxide (O_2_^−^) produced by the host. The observations that the *C. burnetii* SodC is expressed by *C. burnetii* and localizes towards the periphery supports the hypothesis that this enzyme functions in a low pH environment and may play a role in protecting *C. burnetii* from exogenously produced reactive oxygen intermediates.

It has been well established that *C. burnetii* is an acidophile [19-21] and although the cytoplasmic pH of the organism remains near neutral [22], the periplasmic space is presumably acidic. Therefore, we hypothesized that a periplasmically localized Cu/ZnSOD in *C. burnetii* would be active at low pH to defend the organism from phagocyte derived O_2_^−^. To test this hypothesis, the ability of purified, recombinant *C. burnetii* Cu/ZnSOD to inhibit cytochrome C reduction at low pH was determined. The recombinant Cu/ZnSOD did retain activity at a pH of 5.0 suggesting that indeed this enzyme could function in the periplasmic space and protect *C. burnetii* from host derived O_2_^−^. Interestingly, unlike acid phosphatase and catalase activity previously demonstrated for *C. burnetii*, we were not able to detect optimal enzymatic activity at any of the three pH values tested for the CuZnSOD. Maximal catalase activity was detected at pH 7.0 with much lower activity detected at pH 4.5, whereas optimum acid phosphatase activity was observed at pH 5.0 and significantly decreased as the pH was raised [1,3]. Bovine Cu/ZnSOD for example has been shown to retain activity in 8.0 M urea or 2% SDS and exhibit essentially constant activity over the pH range 5.0–9.5 [23-25]. However, *E. coli* Cu/ZnSOD is very thermolabile and sensitive to pH [10]. Thus, whether or not *C. burnetii* Cu/ZnSOD does have a pH optimum requires further study, but our data clearly demonstrates that this enzyme is active at low pH.

Standard methods such as targeted gene disruption, antibiotic selection and growth on axenic media used to manipulate free living bacteria have not been readily available for *C. burnetii*. Although the genetic transformation of *C. burnetii* was first reported in 1996 [26] and later in 2009 [27] along with the ability to grow *C. burnetii* axenic media [28], performing targeted mutagenesis remains challenging. This inability to readily manipulate *C. burnetii* genetically has led to the use of heterologous cloning as a means to study the organism’s genes and regulatory functions. *C. burnetii* genes such as *dnaJ, era, pyrB, sdhCDAB, icd, rnc,* and *SodA/SodB* were used to successfully complement paralogous gene mutations in *E. coli* [2,29-34]. To test functional expression of the *C. burnetii* Cu/ZnSOD, pREB102 was transformed into an *E. coli sodC* mutant (AS454) that was previously demonstrated to be highly susceptible to H_2_O_2_ killing in stationary phase [8]. Over expression of the *C. burnetii sodC* restored resistance of the *E. coli sodC* mutant to H_2_O_2_ killing to wild type levels. Immunoblot analysis confirmed that expression of *C. burnetii sodC* in AS454 was achieved only upon induction, suggesting that the restored resistance of the complemented mutant was not due to an artifact of pREB102 or some unknown factor (data not shown). This data suggests that this enzyme does possess antioxidant properties and supports the hypothesis that this enzyme may play a role in *C. burnetii* intracellular survival in an oxidative stress environment.

## Conclusions

In conclusion, we have demonstrated that *C. burnetii* does express a Cu/ZnSOD that is functional at low pH, which appears to be excreted, and was able to restore H_2_O_2_ resistance in an *E. coli sod*C mutant. These studies provide the framework to evaluate the role that *C. burnetii* SodC plays in intracellular survival. To address this issue, the potential for regulation of this enzyme during oxidative stress and/or by RpoS is currently under investigation, which should provide insight about the possible role of this enzyme in a developmental life cycle and virulence.

## Methods

### Bacterial strains, plasmids, media, and growth conditions

Bacterial strains (*C. burnetii*, and *E. coli*) and plasmids used in this study are listed in Table 1. Luria-Bertani (LB) medium was purchased from Difco Laboratories (Detroit, Mich.) *E. coli* bacteria were cultured aerobically at 37°C. Antibiotics, when required, were incorporated into the culture media or plates at the following concentrations: ampicillin, 100 mg/liter; carbenicillin, 50 mg/liter, and chloramphenicol, 20 mg/liter.

**Table 1:** **List of bacterial strains and plasmids**

**Strain or plasmid**	**Description**	**Reference (s) or source**
*C. burnetii*	Nine mile, phase I, RSA 493	
*E. coli*		
DH5α		Invitrogen
TOP10		Invitrogen
AN387	F^−^, rpsL, gal	(8)
AS454	AN387, *sodC*::spec	(8)
Plasmids		
pBAD-TOPO	TA cloning vector Ap^r^	Invitrogen
pREB102	pBAD-TOPO with 570 bp *C.burnetii sodC* insert	This work

### Cloning, expression, and purification of rCbSodC

PCR amplification of *C. burnetii sodC* was performed in a Biometra UNO-Thermoblock (Biometra, Tampa, Fla.). Primers FBB1.2 (5’-GGAAATATTTTGAGGCGCGTC-3’) and RBB1.2 (5’-ACACGCAATTCGCGCACC-3’) **(**Sigma Genosys, Woodlands, Tx.) were used at final concentrations of 0.2 mM to amplify a 570 bp fragment including the signal sequence of the *C. burnetii sodC* gene. Amplification consisted of an initial 2 min denaturation step at 94°C followed by 30 cycles of 30 s at 94°C, 30 s at 60°C, and 1 min at 70°C. PCR products were visualized in a 1.2% agarose gel. The PCR product was then directly cloned into pBAD-TOPO (Invitrogen) and transformed into TOP10 cells. Restriction digests were performed on the constructs with *Bst*EII to determine which clones contained the insert in the correct orientation. Plasmid DNA from one of those constructs was sent to the Gene Technologies Laboratory at Texas A & M University for sequencing. This construct was designated pREB102.

Expression of the recombinant SodC peptide was done using the pBAD-TOPO expression system (Invitrogen). Briefly, 10 ml of an overnight culture of TOP10 (pREB102) was inoculated into 1 liter of LB broth containing ampicillin (100 μg/ml) and incubated until an OD_600_ of 0.5 was reached. Induction was achieved by adding 9 ml of 2% arabinose and incubating the culture for 4 hr at 37°C on a shaker. Cultures were centrifuged at 5,000 rpm for 10 min. Purification of the recombinant SodC protein was then done using a 1 ml HiTrap nickel affinity column (Amersham Biosciences, Piscataway, NJ.). Briefly, the pellet was resuspended in 50 ml of binding buffer (0.02 M NaPO_4_, 0.5 M NaCl) pH 7.4 containing 50 mg of lysozyme and incubated on ice for 30 min. Cells were lysed using a French press and then centrifuged for 15 min at 5,000 rpm. Twenty milliliters of the French press lysate was filtered through a 0.8/0.45 μm syringe filter and loaded onto the HiTrap nickel column using a parastaltic pump. The column was washed with 8 ml of binding buffer and then washed with 4.5 ml of wash buffer (binding buffer with 100 mM imidazole). His-tagged recombinant protein was eluted using elution buffer (binding buffer with 0.5 M imidazole and collected in 0.1 ml fractions and frozen at-20°C until SDS-PAGE and Western blot analysis.

### Superoxide dismutase activity gels

SodC activity was visualized using a method previously described by Beauchamp *et al.* [16] incorporating the modification of Steinman [17]. Briefly, after electrophoresis on 12% native PAGE, gels were soaked in a riboflavin solution (0.028 M TEMED, 2.8 x 10^5^ M riboflavin, 0.036 M KPO_4_ pH 7.8 for 20 min. at 37°C in the dark followed by soaking them in 0.2% nitroblue tetrazolium (NBT) for 10 min. at 37°C in the dark. The gels were then illuminated for approximately 30 min on a transilluminator. SOD activity corresponds to achromatic zones in a uniformly purple background. To identify the copper zinc nature of the cloned *C. burnetii* SOD, one of the gels was soaked in 5 mM hydrogen peroxide (H_2_O_2_) for 1 hr prior to staining.

### Superoxide dismutase assay

The copper-zinc nature of the recombinant *C. burnetii* SOD was confirmed using a superoxide dismutase assay kit from Cayman Chemical. The assay is based on the utilization of tetrazolium salt for the detection of superoxide anions generated by xanthine oxidase and hypoxanthine. The activity of SODs is determined by their ability to inhibit the reaction. Approximately one unit of each SOD was utilized in the assay. One unit is defined by the amount of SOD that will inhibit the reaction by approximately 50%. The detection of superoxide dismutase activity was done following the manufacturer’s instructions. Specific inhibition of the Cu/Zn SOD activity was achieved by treating the control Cu/Zn SOD and recombinant *C. burnetii* SOD with 1 mM DDC for 15 min. at room temperature prior to superoxide dismutase activity detection. Manganese (Mn) and Iron (Fe) co-factored SODs from Sigma were included to demonstrate the specificity of DDC for Cu/Zn co-factored SODs. SOD assay kit sample buffer was used to dissolve and dilute all SODs and was included in the no SOD control reactions.

### pH effect on the activity of recombinant *C. burnetii* SodC

The activity of the recombinant SodC was determined at pH 5.0, 7.0, and 9.0 by the method described by Flohè and Ötting [35] with some modifications. Briefly, 10 μg purified rSodC was added to 50 mM sodium acetate buffer pH 5.0, 50 mM potassium phosphate buffer pH 7.0, or 50 mM Tris–HCl buffer pH 9.0, respectively, each containing 0.01 mM EDTA. Prior to absorbance readings, 20 μM cytochrome c from horse heart (Sigma-Aldrich), 4 μl of 25 mM hypoxanthine (Calbiochem) and xanthine oxidase from bovine milk (Calbiochem). For control reactions, the hypoxanthine and xanthine oxidase were added in the presence of the rSodC elution buffer. The amount of xanthine oxidase added to each buffer system was adjusted to achieve a reduction rate of approximately 0.0125 absorbance/min at 550 nm. The final reaction volume was 100 μl per well in 96 well plates. Plates were incubated at 37°C and reduction was monitored for 10 min by measuring absorbance at 30 sec intervals. Inhibition of cytochrome C reduction was determined by dividing the absorbance values at 10 minutes with rSodC by the absorbance values at 10 minutes without rSodC and multiplying by 100.

### *sodC* expression by *C. burnetii*

Purified recombinant *C. burnetii* SodC (rSodC) was combined with the adjuvant Titermax (Sigma) and used to immunize a rabbit for the production of monospecific polyclonal antibodies against rSodC. To determine whether or not a CuZnSOD is expressed by *C. burnetii*, *C. burnetii* cells purified from persistently infected L929 murine fibroblasts or *E. coli* cells overexpressing rSodC were suspended in sample buffer (4% SDS, 10% β-mercaptoethanol, 20% glycerol, and 0.25 M Tris, pH 8.0), boiled for 10 min, separated on 15% SDS-PAGE gels, and transferred to nitrocellulose membranes (Biorad, Hercules, CA). Membranes were blocked with 10% nonfat powdered milk and 0.2% Tween-20 in Tris buffered saline, pH 7.4. Blots were then incubated with rabbit antiserum to rSodC at a 1:2,000 dilution followed by incubation with goat anti-rabbit IgG horseradish peroxidase conjugated secondary antibody at a 1:5,000 dilution (Biorad). The blots were developed using an enhanced chemiluminescence system with luminol substrate (Amersham Biosciences). Images were visualized using Kodak Scientific Imaging film.

### Immunogold transmission electron microscopy

L929 murine fibroblast cells persistently infected with *C. burnetii*, Nine Mile, (RSA493), were fixed and processed as described previously [36]. Cells were fixed with 0.2% picric acid, 1% glutaraldehyde, 4% paraformaldehyde, 0.5 mM CaCl_2_ in phosphate buffered saline (PBS, 140 mM NaCl_2_, 3 mM KCl_2_, 2 mM KPO_4_, 10 mM NaPO_4_), pH 7.4 for 3 hr at room temperature while turning end over end. Cells were spun at 10,000 x g for 10 min and the pellet was incubated for 1 hr at 4°C after resuspension in 50 mM NH_4_Cl, 250 mM sucrose, PBS. Cells were then centrifuged at 10,0000 x g for 10 min. Ammonium chloride was removed by resuspending the pellet in 3.5% sucrose, 0.5 mM CaCl_2_ in PBS pH 7.4 overnight at 4°C. Phosphate buffers were removed by washing 4 x 15 min with 0.1 M maleate buffer, with 3.5% sucrose, pH 6.5. Post fixation staining was carried out with 2% uranyl acetate in sucrose/maleate buffer, pH 6.0 for 2 hr at 0°C protected from light. Dehydration and infiltration into LR White was carried out a room temperature in 45 min. steps of: 50% acetone, 70% acetone, 90% acetone, 1:1 100% ethanol/LR White, 3:7 100% ethanol/LR White, 100% LR White, then fresh LR White overnight, second change of fresh LR White before samples were enclosed in gelatin capsules (Electron Microscopy Scences, Hatfield, PA). Polymerization was carried out at 50°C for 24 hr. Silver to gold sections were collected on 300 mesh nickel grids (Electron Microscopy Sciences). All staining was carried out in the BioWave microwave (Ted Pella, Inc., Redding, CA.) at 30°C. Blocking (2% nomal goat sea), primary (rabbit anti-sodC, 1:25 dilution in 2% normal goat sera), and secondary antibody incubations (goat anti-mouse conjugate 12 nm gold particles, (Jackson ImmunoResearch laboratories, West Grove, PA.), diluted 1:40 in 2% normal goat sera were done in three cycles of 1 min on, 1 min off at full power. Primary antibody was washed three times in Tris buffered Saline (TBS, 5 mM Tris, 15 mM NaCl_2_), pH 7.4, for one min per wash. Secondary antibody was washed in TBS pH 8.4, 3 x 1 min. Secondary antibody was fixed in 1% glutaraldehyde for 1 min with gentle agitation at room temperature. Fixed grids were briefly washed with water then stained with 2% uranyl acetate in the microwave for six seconds at full power. Grids were briefly washed in water with gentle agitation at room temperature. Grids were viewed with a JEOL operated at 100 kV.

### Complementation of *E. coli sod*C null mutant

Complementation studies were carried out using an *E. coli sodC* mutant (AS454) previously demonstrated to be more sensitive to killing by exogenous H_2_O_2_ than the wild type parental strain (AN387) during early stationary phase [8]. To genetically complement the *sodC* mutant, plasmid pREB102 was electroporated into AS454. Transformants were selected on LB agar plates containing ampicillin (150 μg/ml). AN387, AS454, and AS454 (pREB102) were grown in LB broth or LB broth containing ampicillin (100 μg/ml) overnight at 37°C on a shaker, subcultured into fresh media at a starting OD_600_ of ≈ 0.01. In order achieve expression of *sod*C, AS454 and AS454 (pREB102) were either induced or not induced with 2% arabinose 4 hr prior to H_2_O_2_ challenge. At 45 min intervals aliquots for H_2_O_2_ challenges were removed, diluted 1:10,000 in PBS, and challenged with 2 mM H_2_O_2_ for 30 min while shaking at 37°C as previously described [8]. Survival was determined as the percentage of colony counts (cfu/mL) from surviving bacteria after H_2_O_2_ treatment and from untreated bacteria by plating on LB plates with or without ampicillin (150 μg/ml).
